# Long-term changes in brain cholinergic system and behavior in rats following gestational exposure to lead: protective effect of calcium supplement

**DOI:** 10.1515/intox-2015-0025

**Published:** 2015-12

**Authors:** Chand D. Basha, Rajarami G. Reddy

**Affiliations:** Department of Zoology, Sri Venkateswara University, Tirupati - 517502, India

**Keywords:** lead toxicity, calcium supplementation, long-term effects, cholinergic system, behavior, blood Pb levels

## Abstract

Our earlier studies showed that lactational exposure to lead (Pb) caused irreversible neurochemical alterations in rats. The present study was carried out to examine whether gestational exposure to Pb can cause long-term changes in the brain cholinergic system and behavior of rats. The protective effect of calcium (Ca) supplementation against Pb toxicity was also examined. Pregnant rats were exposed to 0.2% Pb (Pb acetate in drinking water) from gestational day (GD) 6 to GD 21. The results showed decrease in body weight gain (GD 6–21) of dams, whereas no changes were observed in offspring body weight at different postnatal days following Pb exposure. Male offspring treated with Pb showed marginal alterations in developmental landmarks such as unfolding of pinnae, lower and upper incisor eruption, fur development, eye slit formation and eye opening on postnatal day (PND) 1, whereas significant alterations were found in the righting reflex (PNDs 4–7), slant board behavior (PNDs 8–10) and forelimb hang performance (PNDs 12–16). Biochemical analysis showed decrease in synaptosomal acetylcholinesterase (AChE) activity and an increase in acetylcholine (ACh) levels in the cortex, cerebellum and hippocampus on PND 14, PND 21, PND 28 and in the four-month age group of rats following Pb exposure. Significant deficits were also observed in total locomotor activity, exploratory behavior and open field behavior in selected age groups of Pb-exposed rats. These alterations were found to be maximal on PND 28, corresponding with the greater blood lead levels observed on PND 28. Addition of 0.02% Ca to Pb reversed the Pb-induced impairments in the cholinergic system as well as in behavioral parameters of rats. In conclusion, these data suggest that gestational exposure to Pb is able to induce long-term changes in neurological functions of offspring. Maternal Ca administration reversed these neurological effects of Pb later in life, suggesting a protective effect of calcium in Pb-exposed animals.

## Introduction

Exposure to lead (Pb) has long been known to exert toxic effects on the nervous system with the greatest concern in general for unborn fetuses, infants and young children. Developmental exposure to Pb has become an important public health concern because of the possible toxic impact on sensitive development and programing of organ functions (Needham *et al.,* 2011). Recent developments suggest that gestation through lactation is a sensitive period when exposure to Pb (Kim *et al.,* 2002) causes long-lasting deficits in brain and neurobehavioral functions (Bihaqi *et al.,* 2014). However, most of these studies investigated late life health effects of Pb following post-weaning exposure. It is not known whether gestation-only Pb exposure is sufficient enough to induce long-lasting deficits in neurobe-havioral functions. Maternal Pb exposure has a significant influence on embryonic and fetal development (Gargouri *et al.,* 2012). Pb readily crosses the placental-fetal barrier, causing a direct relation between the Pb exposed mother and the possibility of irreversible developmental damage to offspring (Osman *et al.,* 2000; Khoradad *et al.,* 2013). Studies have also shown equivalent cord blood Pb levels compared with maternal blood Pb levels (Raghunath *et al.,* 2000). Another study, Tellez-Rojo *et al.,* (2004), reported that the Pb concentration in fetal and infant blood influenced by that in maternal blood during pregnancy resulted in long-term Pb deposits in bones. Pb exposure during pregnancy is associated with a variety of neonatal perturbations, including low birth weight, delayed development and maturation, as well as later developing behavioural outcomes (Chang *et al.,* 2012). Experimental studies demonstrated that administration of Pb to pregnant rats produced destructive changes in the brains of their offspring (Lebed'ko & Ryzhavskii, 2005; Ryzhavskii *et al.,* 2008). Differential gender effects were also observed after *in utero* exposure to Pb throughout full gestation (Cory-slechta *et al.,* 2013). Wirbisky *et al.,* (2014) reported that Pb adversely affected the GABAergic system and altered the function of different genes during embryogenic development in zebrafish. Our previous studies also suggested that Pb exposure during lactation or gestation caused irreversible long-lasting deficits in the cholinergic and aminergic systems and in the behavior of rats (Reddy *et al.,* 2003; Prasanthi *et al.,* 2010; Basha *et al.,* 2012).

Maternal calcium (Ca) is an essential mineral for the newborn infant and alteration in Ca signaling may have life-long consequences in brain functions (Bass *et al.,* 2006; Cunnane *et al.,* 2014). It has been reported that Ca homeostasis in neurons can be disrupted by Pb exposure, exhibiting excitotoxic effect on synaptic function (Han *et al.,* 2000). Inadequate Ca consumption was shown to increase Pb absorption and Pb retention, suggesting that the Ca status may be an important factor in the maternal-fetal transfer of Pb across the placenta (Saleh *et al.,* 2011). Several studies reported that adequate dietary Ca intake supported normal fetal growth, improved cognitive and motor functions and also reduced the Pb exposure to the developing fetus by reducing blood Pb (PbB) levels during the development of the rat (Schwartz *et al.,* 2007; Keating *et al.,* 2011). Our previous studies have suggested that Ca supplementation could reverse the Pb-induced neurochemical changes during development (Prasanthi *et al.,* 2010; Gottipolu *et al.,* 2014, Basha *et al.,* 2014). In the present study, we examined the protective effect of calcium supplementation against maternal Pb exposure-induced long-term effects on the development of the offspring, on the brain region specific cholinergic system and on behavioral functions in rats.

## Materials and methods

### Chemicals

Lead acetate (99.99% purity) was purchased from Sigma Chemicals (St. Louis, MO, USA) and all other chemicals from Merck, India.

**Figure 1:**
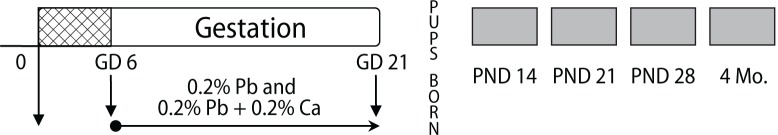
Study design: A schematic representation of the exposure period and the parameters determined in different age groups of rats.

### Animal exposure

Female and male albino rats (Wistar) were acclimatized for one week before mating. Two females and one male were placed overnight in a cage and the presence of a vaginal sperm plug was recorded. Pregnancies determined by the presence of sperm in the vaginal smear were considered gestational day (GD) 1. On GD 6, the dams were randomly assigned to three experimental groups (Group I – control, Group II – 0.2% Pb (lead acetate) exposed rats and Group III – animals exposed to 0.02% calcium supplement together with Pb. The animals were housed individually. Pregnant animals were exposed to low level Pb (0.2%) by intake of Pb-acetate dissolved in deionized water from gestational day 6 (GD 6) until the birth of pups (GD 21) (Fig. 1). Calcium was supplemented as 0.02% in Pb-water (calcium chloride dissolved in distilled water containing Pb) (Fig. 1). Pb water together with calcium was given to the dams from GD 6 until the pups were born (GD 21). Control animals received only deionized water without Pb. All pups in each experimental group were pooled 24 h after birth (PND1) and the new litters consisting of eight males were randomly selected and placed with each dam. The animals were housed at constant temperature (28±2 °C) and relative humidity 60±10% with a 12 h light/dark cycle. Standard rat chow (Sri Venkateswara Traders, Bangalore, India) and water were made available ad libitum. The protocol and animal use were approved by the Animal Ethical Clearance Committee, S.V. University. The exposure dose levels of Pb and calcium were established in our earlier published works (Basha *et al.,* 2003; Reddy *et al.,* 2007; Prasanthi *et al.,* 2010; Basha *et al.,* 2014).

### Developmental landmark studies

Starting with PND 1, the number of pups (alive and dead) was counted and gender differences were recorded. The male pups were weighed and observed for developmental signs, such as fur development (the appearance of fur sufficient to cover skin), incisor teeth eruption (the first appearance of the upper and lower incisors), pinnae detachment (both ears completely unfolded from the head), eye slit formation, eye opening (both eyes fully open) and crown rump length (De Castro *et al.,* 2007; Raffaele *et al.,* 2010).

### Survival index of pups

The survival index of pups on PND 4 and PND 21 was calculated using the following formulas (De Castro *et al.,* 2007; Raffaele *et al.,* 2010):

- 4^th^ day survival index: [Number of live offspring at lactation day 4/Number of live offspring delivered x 100],
- 21^st^ day survival index: [Number of live offspring at lactation day 21/Number of live offspring delivered x 100]

### Body weight of dams and offspring

The differences in body weight of dams were recorded on GD 6 and GD 20 and those of the male offspring were recorded on PNDs 1, 7, 14, 21, 28 and in 4-month-old control and experimental rats.

### Early postnatal behavioral studies

During the lactation period, male pups of each dam were evaluated for reflex development and neuro-muscular maturation. All pups/litter were removed from the home cage and placed in a small holding cage prior to testing (Ferguson *et al.,* 2001; Garey *et al.,* 2005).

### Righting reflex (PNDs 4–7)

Each pup was placed with the dorsal side down on a smooth flat surface and the latency to the position on all four paws was recorded over a period of 60 s duration. One trial/day was used for each of the test days (Ferguson *et al.,* 2001; Garey *et al.,* 2005).

### Slant board behavior (PNDs 8–10)

Each pup was placed on the ventral side down, with its head pointing toward the lower end on a wood board angled at 45° horizontally. Latency to turn 180° from the original starting position was measured allowing a maximum of 60 seconds. Each pup was tested for a single trial on each of the three test days (Ferguson *et al.,* 2001; Garey *et al.,* 2005).

### Forelimb hang (PNDs 12–16)

Each pup closely posed to a string (41 cm above a padded surface) stretched between two wooden blocks was then permitted to grasp the string with its forepaws, where the point was released, so that it was suspended from the string by its forepaws. The latency to fall from the string (maximum 60 s) was measured and each pup was tested for a single trial on each of the five test days (Ferguson *et al.,* 2001; Garey *et al.,* 2005).

### Acetylcholine (ACh) determination

The acetylcholine content was determined by the method of Metcalf (1951) as given by Augustinson (1963). The synaptosomal fractions of the hippocampus and cerebellum were placed in boiling water for 5 minutes to terminate AChE activity and also to release the bound ACh. To the synaptosomal fractions, 1 ml of alkaline hydroxylamine hydrochloride followed by 1 ml of 50% hydrochloric acid was added. The contents were mixed thoroughly and centrifuged. To the supernatant, 0.5 ml 0.37 M ferric chloride solution was added and the brown color developed was read at 540 nm against a reagent blank in a spectrophotometer and expressed as acetylcholine (ACh) content (μmoles of ACh/g).

### Determination of acetylcholinesterase (AChE) activity

AChE specific activity was determined following the method of Ellman *et al.,* (1961). The reaction mixture contained 3.0 ml of phosphate buffer (pH 8.0), 20 μl of 0.075 M acetylthiocholine iodide (substrate) and 100 μl of 0.01 M DTNB (5, 5-dithiobis-2-nitrobenzoic acid). The reaction was initiated with the addition of 100 μl of synaptosomal fraction. The contents were incubated for 30 min at room temperature and the color absorbance was measured at 412 nm in a spectrophotometer (Hitachi, Model U-2000). The enzyme activity was expressed as μmoles of ACh hydrolyzed/mg protein/hr.

### Determination of protein content

Protein content of the tissues was measured by the method of Lowry *et al.,* (1951).

### Postnatal Behavioral Studies

Total locomotor activity: Locomotor activity of control, Pb-exposed, and calcium supplemented rats was measured using OPTO-VARIMEX (Columbus instruments, USA) at the designated time periods (PNDs) for a period of two hours in the morning (08.00–10.00AM). The activity was presented as counts/min.

Open field behavior: The open field behavior was measured in a chamber measuring 90×90×90 cm. The floor of the arena was divided into 36 equal squares. The number of squares crossed with all paws (crossing), standing on the hind legs (rearing), standing on the hind legs and placing the forelimbs on the wall (wall rearing), placing the nose against the wall or floor (sniffing), and wiping, licking or combing of any part of the body (grooming) were recorded. The sum of all tasks in open field was considered the total behavior (Netto *et al.,* 1986).

Exploratory behavior: Exploratory behavior was measured in a box with a hole board bottom (90x90 cm) containing three equally spaced holes (3 cm in diameter) in the floor. Each rat was placed in the center of the arena for 5 min., the time during which the number of head dips and head-dipping duration (in seconds) were recorded. A head dip was scored if both eyes disappeared into the hole.

### Determination of blood Pb levels

For the determination of Pb levels, 5.0 ml of whole blood was taken by heart puncture. After digestion with concentrated nitric acid using a microwave digestion system followed by addition of 30% hydrogen peroxide, the samples were brought to a constant volume. Blood Pb levels were determined with an atomic absorption spectrophotometer (AAS-Shimadzu-AA 6300) with graphite furnace (GFA-EX7i) (Ballentine *et al.,* 1957).

### Analysis of data

The data was subjected to one way analysis of variance (ANOVA) followed by Student-Newman-Keuls (SNK) post hoc test using statistical package for social sciences (SPSS 16) to compare the effects among various groups. The 0.05 level of probability was used as the criterion for significance. All the data in this study were analyzed using the dam or litter as the experimental unit. For some behavioral measures, two pups from each litter were tested, but the values were averaged and only one value from each litter was considered for statistical analysis. The variation within the litter was less than 5%.

## Results

**Figure 2:**
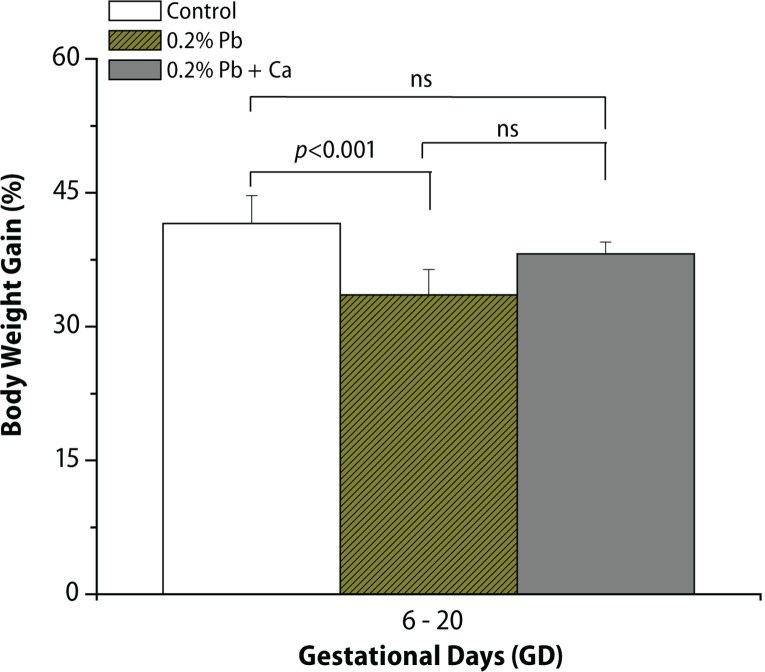
Effect of Pb-exposure and calcium supplementation on maternal body weight gain (%) of rats. Rats were exposed to either deionized drinking water (control) or Pb-acetate (0.2%) or calcium together with Pb in deionized water from gestational day 6 (GD 6) to GD 21 through drinking water. Values are mean ± SD of eight (n=8) observations. The values marked with “asterisk” are significantly different from corresponding controls as evaluated by the ANOVA followed by Student-Newman-Keuls (SNK) post hoc test (*p*<0.05).

**Figure 3:**
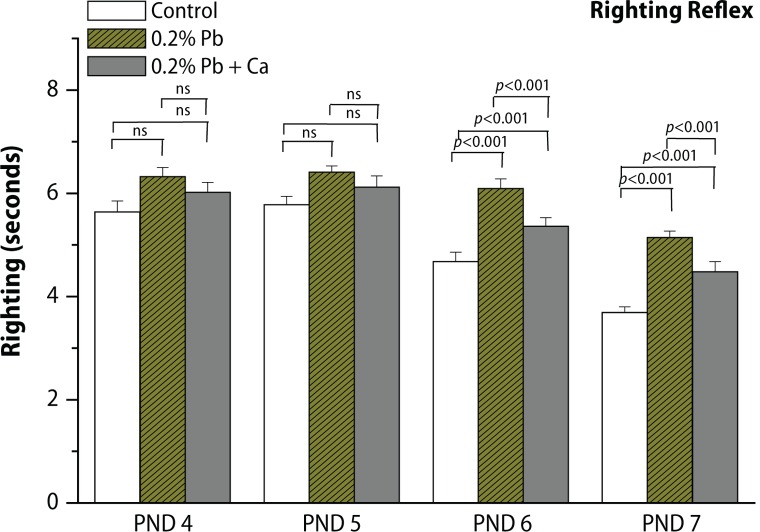
Effect of Pb-exposure and calcium supplementation on righting reflex (time in seconds) behavior in rats. Rats were exposed to either deionized drinking water (control) or Pb-acetate (0.2%) or calcium together with Pb in deionized water from gestational day 6 (GD 6) to GD 21 through drinking water. Values are mean ± SD of eight (n=8) observations. The values marked with “asterisk” are significantly different from corresponding controls as evaluated by the ANOVA followed by Student-Newman-Keuls (SNK) post hoc test (*p*<0.05).

**Figure 4:**
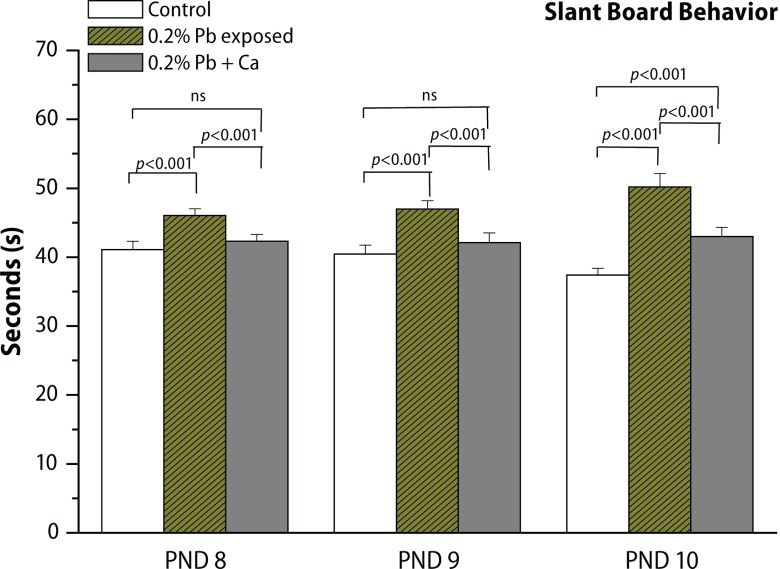
Effect of Pb-exposure and calcium supplementation on slant board behavior (time in seconds) of rats. Rats were exposed to either deionized drinking water (control) or Pb-acetate (0.2%) or calcium together with Pb in deionized water from gestational day 6 (GD 6) to GD 21 through drinking water. Values are mean ± SD of eight (n=8) observations. The values marked with “asterisk” are significantly different from corresponding controls as evaluated by the ANOVA followed by Student-Newman-Keuls (SNK) post hoc test (*p*<0.05).

**Figure 5:**
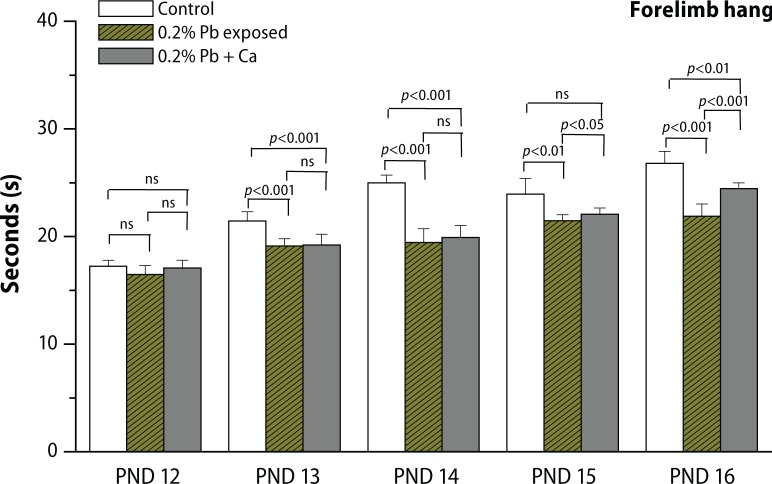
Effect of Pb-exposure and calcium supplementation on forelimb hang performance (time in seconds) of rats. Rats were exposed to either deionized drinking water (control) or Pb-acetate (0.2%) or calcium together with Pb in deionized water from gestational day 6 (GD 6) to GD 21 through drinking water. Values are mean ± SD of eight (n=8) observations. The values marked with “asterisk” are significantly different from corresponding controls as evaluated by the ANOVA followed by Student-Newman-Keuls (SNK) post hoc test (*p*<0.05).

In the present study, we evaluated the protective effect of calcium supplement against Pb-induced effects on the development of offspring and neurobehavioral functions in different postnatal age groups of rats. The rats were observed daily for the development of clinical signs of toxicity throughout the treatment period. No mortality and abnormal behavioral patterns were observed either in control or in treated pregnant rats. Pb exposure significantly decreased the maternal body weight gain recorded from GD 6 to GD 20 (*p*<0.05), while the body weight of male offspring from Pb-exposed mothers showed no significant changes on PND 7, PND 14, PND 21, PND 28 and in 4-month-old rats compared to controls (Figure 2, Table 3). Analyses of litter number/dam, male fetal body weight and crown rump length were done on PND 1. No significant changes were observed in external morphology of fetuses following Pb exposure (Table 1). Beginning with PND 1, all retained male pups were observed daily for the occurrence of developmental landmarks, such as unfolding of pinnae, fur development, lower and upper incisor eruption, eye slit formation and eye opening (Table 2), but no significant effects of Pb were found in these developmental landmarks. Early postnatal neurobe-havioral studies such as righting reflex (PND 4 to PND 7), slant board behavior (PND 8 to PND 10) and forelimb hang performance (PND 12 to PND 16) were observed in control and treated rats (Figures 3–5). Significant increase (*p*<0.01) was observed in the latency to turn in the righting reflex and slant board behavior of Pb-exposed offspring (Figures 3 and 4). Pb exposure also decreased the forelimb hang performance (*p*<0.01) recorded from PND 12 to PND 16 (Figure 5). Significant recovery was observed in the development of offspring and neuromotor maturation when Pb was supplemented with calcium (Figures 2–5; Tables 1 and 2).

**Table 1:** Effect of calcium supplementation on Pb induced alterations in the external morphology of fetuses of dams of control and experimental rats.

Parameters	Control	0.2% Pb	0.2% Pb + Ca
Litter number/dam	9.62±1.7^a^	8.75±1.2^a^ (-9.04)	8.87±1.4^a^ (-7.79)
Male fetal body weight (g)	5.85±0.5^a^	5.63±0.4^a^ (-3.7)	5.75±0.5^a^ (-1.7)
Crown rump length (mm)	53.27±0.7^a^	51.57±0.6^a^ (-3.1)	52.88±0.8^a^ (-0.73)

a Values are mean ± SD of 8 observations. Values in the parentheses are percentage change from control. Values not sharing a common superscript (^a,b^)differ significantly at *p*<0.05.

**Table 2:** Effect of calcium supplementation on Pb induced alterations of developmental landmarks in rats.

Parameters	Control	0.2% Pb	0.2% Pb + Ca
Pinna unfolding (PND)	4.33±0.4^a^	4.41±0.2^a^ (1.84)	4.47±0.3^a^ (3.23)
Lower incisor eruption (PND)	3.2±0.4^a^	3.15±0.4^a^ (1.56)	3.26±0.5^a^ (1.87)
Fur development (PND)	6.62±0.7^a^	6.83±0.5^a^ (3.17)	6.66±0.7^a^ (0.6)
Upper incisor eruption (PND)	9.2±0.4^a^	9.11±0.2^a^ (-0.98)	9.3±0.4^a^ (1.08)
Eye slit formation (PND)	9.95±0.7^a^	9.72±0.6^a^ (-2.31)	9.93±0.6^a^ (0.2)
Eye opening(PND)	12.04±0.5^a^	12.13±0.6^a^ (0.74)	12.02±0.7^a^ (0.16)

a Values are mean ± SD of 8 rats; pups within the litter were pooled, but litter was considered as n of one for statistical analysis. Values in the parentheses are percentage change from control. Values not sharing a common superscript (^a,b,c^) differ significantly at *p*<0.05.

**Table 3:** Effect of calcium supplementation on Pb induced alterations in body weight of male offspring of control and experimental rats.

Parameters	Control	0.2% Pb	0.2% Pb + Ca
PND 7	11.4±1.1^a^	10.8±1.4^a^ (–5.2)	11.1±1.1^a^ (–3.0)
PND 14	16.4±0.5^a^	15.2±1.5^a^ (–7.3)	15.8±0.9^a^ (–3.6)
PND 21	31.2±1.6^a^	29.8±2.1^a^ (–4.4)	30.3±2.3^a^ (–2.8)
PND 28	48.1±2.3^a^	47.1±1.8^a^ (–2.5)	45.3±1.6^a^ (–5.8)
4 Months	261.2±3.5^a^	253.6±2.9^a^ (–2.9)	260.8±4.1^a^ (–0.15)

a Values are mean ± SD of 8 observations. Values in the parentheses are percentage change from control. Values not sharing a common superscript (^a,b^) differ significantly at *p*<0.05.

**Figure 6:**
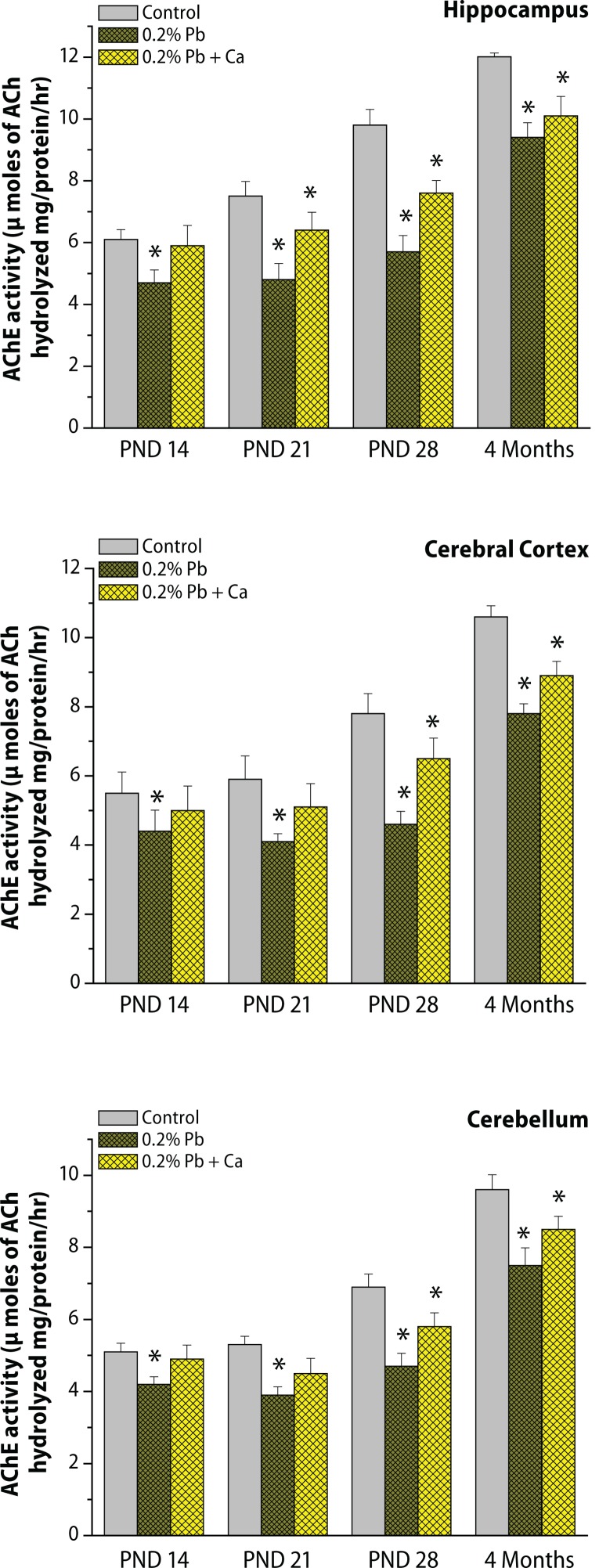
(a,b,c) Effect of Pb-exposure and calcium supplementation on AChE activity in cortex, cerebellum and hippocampus. Rats were exposed to either deionized drinking water (control) or Pb-acetate (0.2%) or calcium together with Pb in deionized water from gestational day 6 (GD 6) to GD 21 through drinking water. AChE activity was determined in the synaptosomal fractions of brain regions. Values are mean ± SD of six separate experiments. The 0.05 level of probability was used as the criterion for significance.

**Figure 7:**
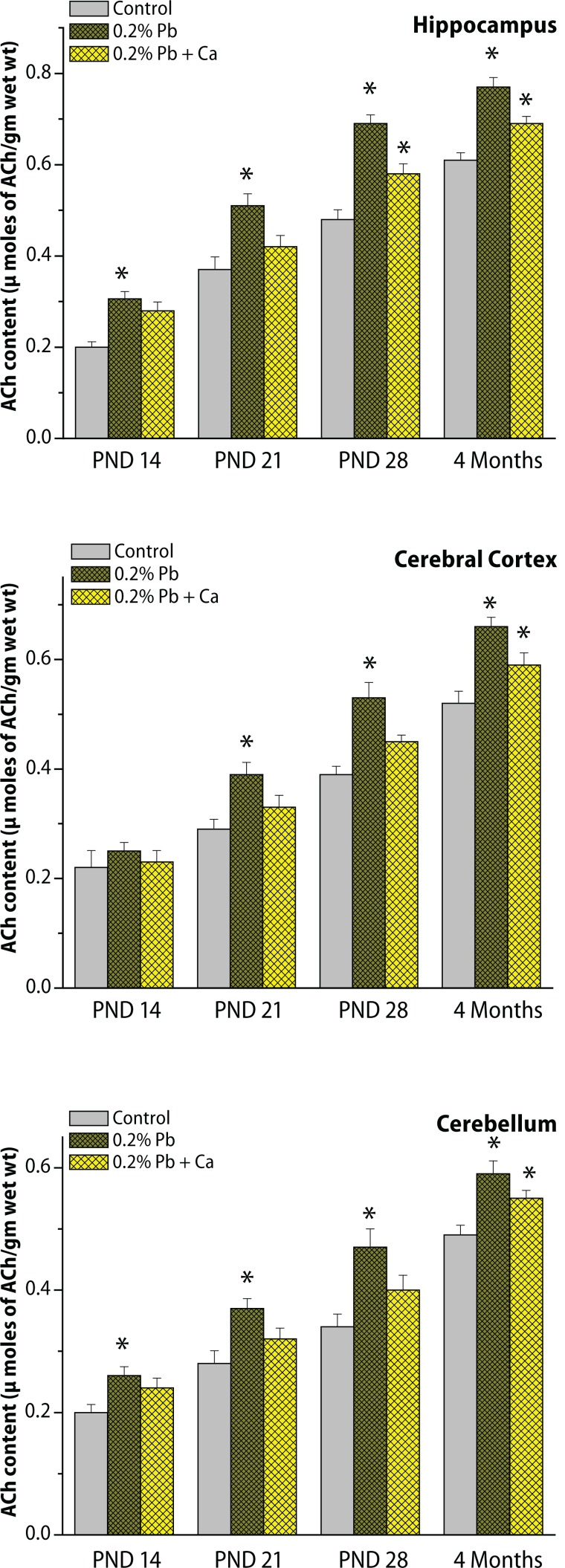
(a,b,c) Effect of Pb-exposure and calcium supplementation to Pb on ACh levels in cortex, cerebellum and hippocampus. Rats were exposed to either deionized drinking water (control) or Pb-acetate (0.2%) or calcium together with Pb in deionized water from gestational day 6 (GD 6) to GD 21 through drinking water. The neurotransmitter levels were determined in the synaptosomal fractions of brain regions. Values are mean ± SD of six separate experiments. The 0.05 level of probability was used as the criterion for significance.

The specific activity of synaptosomal acetylcholinesterase enzyme (AChE) and levels of acetylcholine (ACh) were determined in the cortex, cerebellum and hippocampus on PND 14, PND 21, PND 28 and in the 4-month age groups of control and Pb exposed rats (Figures 6 and 7). As shown in Figures 6 and 7, the activity of AChE and ACh levels increased from PND 14 to 4 months of age. The hippocampus exhibited higher levels of ACh and activity of AChE (Figures 6 and 7) than did the cortex and cerebellum. Exposure to Pb resulted in a decrease in AChE activity and was accompanied by an increase in ACh levels in the cortex, cerebellum and hippocampus at various time points of postnatal development and adult age (alterations in AChE and ACh were respective 36.8% and 34.2% in the hippocampus, 30.5% and 31.9% in the cortex, 26.4% and 32.1% in the cerebellum on PND 21; on PND 28, AChE and Ach amounted to respective 41% and 43.2% in the hippocampus, 39.2% and 41.1% in the cortex, 28.2% and 37.8% in the cerebellum ; at 4 months the respective values were 21.2% and 29.3% in the hippocampus, 26.7% and 24.2% in the cortex, and 21.2% and 24.4% in the cerebellum (Figures 6 and 7). Pb-induced decrease in AChE activity and increase in ACh levels were greater on PND 28 and more pronounced in the hippocampus than in the cortex and cerebellum (Figures 6 and 7). However, Ca supplementation together with Pb reduced the Pb-induced alterations in brain region specific for the cholinergic system in all the selected age groups of rats (Figures 6 and 7).

**Figure 8:**
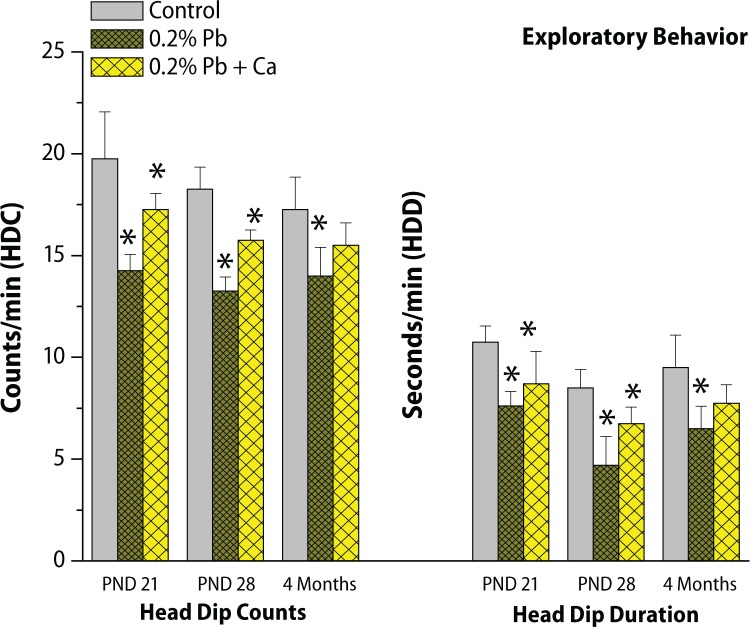
Effect of Pb-exposure and calcium supplementation on exploratory behavior in different age groups of rats. The values are Mean ± SD of six separate experiments. The 0.05 level of probability was used as the criterion for significance.

**Figure 9:**
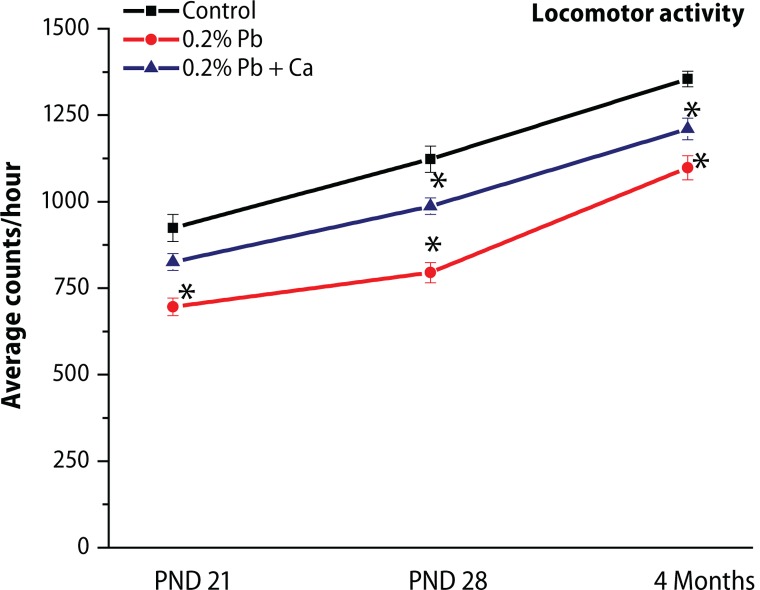
Effect of Pb-exposure and calcium supplementation on total locomotor activity in different age groups of rats. The activity was measured with OPTO-VARIMEX, Columbus Instruments, Ohio, USA on the designated PNDs and was presented as counts/min. The values are Mean ± SD of six separate experiments. The 0.05 level of probability was used as the criterion for significance.

**Figure 10:**
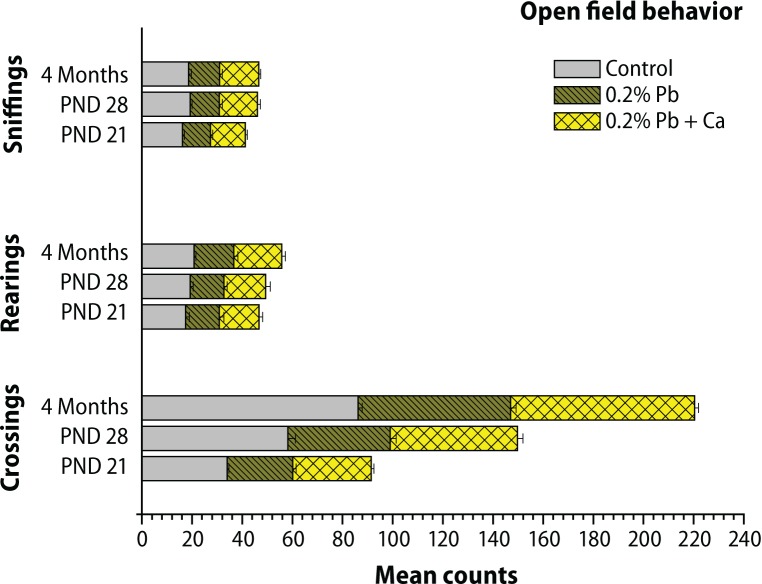
Effect of Pb-exposure and calcium supplementation on open-field behavior in different age groups of rats. The values are Mean ± SD of six separate experiments. The 0.05 level of probability was used as the criterion for significance.

The results of postnatal behavioral studies are presented in Figures 8–10. The open field behavior was evaluated by examining the crossings, rearings and sniffings on PND 21, PND 28 and in the 4-month-old groups of rats. Maternal exposure to Pb decreased the open field behavior, however alterations in sniffings were more prominent compared to crossings and rearings (Figure 8). The exploratory behavior recorded in the three-hole board showed longer head dip duration and higher head dip counts on PND 21, PND 28 and in 4-month-old groups of rats. Significant decrease was observed in head dip counts and head dip duration following Pb exposure in all selected age groups when compared with control rats (Figure 8). Similarly as AChE activity, total locomotor activity showed decrease following Pb exposure in all the age groups of rats (Figure 9). These Pb-induced effects on behavioral studies were greatly reversed with calcium addition to Pb (Figure 10). Blood Pb levels were measured on PND 21, PND 28 and in 4-month-old control and treated rats (Table 4). Pb exposure significantly (*p*<0.001) increased the Pb levels (11.2 μg/dL on PND 21, 12.3 μg/dL on PND 28, and 5.9 μg/dL at 4 months) in all selected age groups of rats (Table 4). The highest Pb levels were observed on PND 28. Addition of calcium to Pb water significantly reduced the Pb levels (35–40%) in all the selected age groups of rats (Table 4).

**Table 4:** Blood Pb levels (μg/dl) in different age groups of control and experimental rats.

Treatments	PND 21	PND 28	4 Months
Control	0.21±0.014^a^	0.33±0.07^a^	0.19±0.04^a^
0.2% Pb	11.2±1.9^b^	12.3±0.9^b^	5.9±0.6^b^
0.2% Pb + Ca	6.7±0.8^c^	7.9±0.62^c^	4.2±0.51^c^

Values are mean ± SD for eight (n=8) rats in each group. Values in parenthe-ses are percentage changes from control.

a,b,c values not sharing a common superscript (a,b,c) differ significantly at *p*<0.05.

## Discussion

The present study was carried out to determine whether gestational Pb exposure would be sufficient to induce late-life neurotoxicity in rats. We further examined how Pb and Ca interacted to influence pregnancy and fetal development. Pregnant women are at high risk for Pb exposure, but the current postnatal screening programs have missed this window of vulnerability (Yang *et al.,* 2003; Virgolini *et al.,* 2006). Recent reports have suggested that Pb exposure during pregnancy causes irreversible injury for mother and fetus (Farag *et al.,* 2010; Modgil *et al.,* 2014). Our results showed significant decrease in body weight gain of Pb exposed dams, whereas no significant changes were observed in male offspring body weight. The loss in body weight of dams can be attributed to decrease in food and water intake or the effect of Pb on the synthesis of active metabolites and to intra-uterine alterations (Canfield *et al.,* 2003; Rezende *et al.,* 2010). Blood Pb levels (PbB) increase approximately by 20% throughout pregnancy, even in women with low blood Pb levels (Bunn *et al.,* 2001; Gulson *et al.,* 2004). Recent studies have reported that PbB levels reach a peak during the second trimester at which point the metal is absorbed by the placenta. This is associated with low birth weight, preterm delivery and abnormal behavior in offspring (Hu *et al.,* 2006; Plusquellec *et al.,* 2007). The morphological assessments made in the current study found no significant differences in litter number, fetal body weight and crown rump length. Neither were other maturational measures used in this study, such as pinnae unfolding, fur development, lower and upper incisor eruption, eye slit formation and eye opening, significantly altered in the offspring exposed to Pb. Han *et al.,* (2000) and Chang *et al.,* (2012) reported that *in utero* exposure to Pb altered birth length, litter size, pup sex and birth weight in male offspring. Several previous studies also reported alterations in eye opening and lower and upper incisor eruption following maternal Pb exposure (Riter *et al.,* 1975; Vorhees, 1985). In contrast, some studies reported no difference in developmental landmarks (Mello *et al.,* 1998; Gandhi and Panchal, 2011). Variability in results of these studies appears to be due to differences in study design and exposure level (Rossi-George *et al.,* 2011; Wirbisky *et al.,* 2014).

Our results showed significant impairment in sensorimotor performance in Pb exposed male pups. The current study was conducted only on male rats to exclude hormonal and reproductive cycle influences which may alter the physiology and metabolism in females. Rapid brain growth occurs during the third trimester in humans, whereas in rats the last week of prenatal life and the first three weeks of postnatal life would be critical for continued neural development and rat pups showed rapid behavioral changes during this period (Clifford *et al.,* 2011). We therefore investigated the effects of gestational Pb exposure on reflex tests which are useful in the assessment of developmental disturbances and also reflect the functioning of various brain regions. We found Pb exposure to cause a significant delay of the righting reflex and slant board behavior, as well as a latency to fall in the grip strength test. Pb-induced alterations in these early postnatal behavioral studies showed variations depending on the developmental timepoint. Earlier observations also showed that Pb-treated animals exhibited deficits in righting reflex and grip strength in rats, suggesting a delay in attaining these skills probably because of damage or poor development of the motor system and brain (Mello *et al.,* 1998). However, previous studies did not show a correlation between the cholinergic system and behavioral abnormalities. As a strong neurotoxic agent, Pb particularly affects the development of the central nervous system (Mansouri, 2013), causing alterations in the cholinergic system (Reddy *et al.,* 2003). We observed a significant decrease in AChE activity and increased in ACh levels in several brain areas including the cortex, cerebellum and hippocampus. Further, we found that the effects of gestational Pb on the cholinergic system were varying, depending both on the given brain region and on age. Our recent studies also established that developmental exposure altered the cholinergic system in different brain regions (Basha *et al.,* 2012; Basha *et al.,* 2014). Another study by Reddy *et al.,* (2007) reported sensitivity of the cholinergic system to Pb, supported by *in vitro* studies of 35 PND rat brains using different concentrations of Pb, which showed dose dependent inhibitory actions on the cholinergic system. However, previous reports found that Pb exposure had a direct action on cholinergic neurons causing decrease of AChE coupled with increased ACh (Mansouri *et al.,* 2013; Phyu & Tangpong, 2013). Pb has a high affinity to free SH groups of AChE and its binding can alter their function, reflecting a possible increase in ACh levels in brain tissues, which is consistent with earlier reports (Reddy *et al.,* 2007; Mansouri *et al.,* 2013; Basha *et al.,* 2014).

The cholinergic system, which is known to influence behavior, is likely to have a role in mediating the changes in emotional behaviors induced by Pb exposure. The present findings clearly showed deficits in the open-field test, exploratory behavior and locomotor activity in Pb-exposed rats. Changes in these behavioral studies can be related to perturbations in the cholinergic system detected in the cortex, hippocampus and cerebellum. However, elevated blood Pb (PbB) levels at any age induce neuro-degeneration that leads to impairment of neurobehavioral functions (Schwartz *et al.,* 2007). Different studies revealed significant associations between PbB levels and alterations in cognitive and motor functions (Payton *et al.,* 1998; Surkan *et al.,* 2008). The present findings also showed that alterations in the neurobehavioral functions persisted in adult rats despite the fact that PbB levels reached normal values. Yang *et al.,* (2003) observed long-term deficits in learning/memory with PbB levels of 40.4 μg/dL on PND 1 and 2.3 μg/dL on PND 65 following exposure to Pb during gestational period. Another study (Bunn *et al.,* 2001) also observed similar PbB levels following gestational Pb exposure. Our results showed that changes in the cholinergic system and neurobehavioral functions remained for a long period of time, even after the end of Pb exposure. Since bone Pb has a half-life of years to decades, women and their infants will continue to be at risk for Pb exposure (Yang *et al.,* 2003; Gulson *et al.,* 2004). The alterations in these region-specific cholinergic systems and behavioral studies were more pronounced and significant on PND 28 than in other age groups of rats. The age-dependent alterations observed postnatally might be due to greater gastrointestinal absorption of Pb in developing animals and also due to an underdeveloped blood-brain barrier (Hu *et al.,* 2011). However, Ca supplementation with Pb significantly reversed the Pb-induced alterations in the maturation of offspring, cholinergic system and neurobehavioral functions in different age groups of rats. Our findings appear to have an important public health concern in light of the high sensitivity of cognitive and motor functions to Pb exposure.

Dietary Ca intake can support normal fetal growth and development during pregnancy in animals and humans (Han *et al.,* 2000). Pb has an affinity to Ca binding sites (Sauk & Somerman, 1991) and Ca deficiency is associated with deficits in the neuro-developmental outcome in young children. Han *et al.,* (2000) have suggested that adequate intake of Ca during pregnancy can reduce fetal Pb accumulation in rats. Previously we also showed that Ca supplementation can reduce Pb-induced developmental neurotoxicity by reversing the alterations in neuro-transmitters and mitochondrial antioxidant enzymes in different brain regions of the rat (Prasanthi *et al.,* 2010; Basha *et al.,* 2012). Dietary Ca intake plays an important role in suppressing mobilization of Pb from maternal bone and /or decreasing gastrointestinal absorption of ingested Pb, thereby decreasing the risk of fetal and infant exposure (Gulson *et al.,* 2004; Ettinger *et al.,* 2009). Adequate Ca intake can compete with Pb in several cellular interactions, reduce the PbB levels, and improve neurobehavioral and motor functions (Tellez-Rojo *et al.,* 2004). Dietary Ca supplementation is a cost-effective means to lower Pb exposure of the developing fetus and the breast feeding infant. Studies have shown that during pregnancy and lactation daily intake of 1,200 mg Ca reduced maternal blood Pb by 15–20% (Han *et al.,* 2000; Ettinger *et al.,* 2009). In the present study, we found that administration of Ca along with Pb reduced the offspring PbB levels by almost 35–40%. Ca supplementation during pregnancy can reduce fetal exposure to Pb, suggesting the therapeutic efficacy of Ca for Pb-toxicity.

## Conclusions

Consistent with the published work and results from our laboratory, it is clear that gestational exposure to Pb is sufficient to induce late life alterations in neurobehavioral functions in rats. These neurobehavioral changes continue even long after the Pb exposure was stopped. Although Ca supplement reversed the Pb-induced neurotoxicity, longterm protection by Ca is partial, suggesting that adequate calcium intake would be beneficial in treating Pb-induced toxicity during pregnancy in rats.
